# Inflammatory pseudotumor of the kidney: a case report

**DOI:** 10.1186/1752-1947-5-411

**Published:** 2011-08-24

**Authors:** Abdelhak Khallouk, Youness Ahallal, Mohammed Fadl Tazi, Hinde Elfatemi, Elmehdi Tazi, Jalaleddine Elammari, Mohammed Jamal Elfassi, Moulay Hassan Farih

**Affiliations:** 1 Department of Urology, Hassan II Teaching Hospital, Fes, Morocco; 2 Department of Pathology, Hassan II Teaching Hospital, Fes, Morocco; 3 Department of Medical Oncology, National Institute of Oncology, Rabat, Morocco

## Abstract

**Introduction:**

Inflammatory pseudotumors, also known as inflammatory myofibroblastic tumors, are uncommon benign tumors of unknown etiology which may develop at several anatomical sites. In the urogenital tract, inflammatory pseudotumor usually affects the urinary bladder or the prostate. Inflammatory pseudotumor of the kidney is very rare. It is considered as a reactive inflammatory lesion that features very good prognosis.

**Case presentation:**

We present the case of a 57-year-old Moroccan man who presented with a two-month history of gross hematuria and left lumbar pain. Imaging investigations revealed a left kidney mass and pathological examination of the nephrectomy specimen showed an inflammatory pseudotumor.

**Conclusion:**

As the preoperative definitive diagnosis of such a tumor is not possible, surgery is advised because only pathological examination of the nephrectomy specimen can establish the diagnosis with certainty. From one case report and literature review, the authors suggest a diagnostic and therapeutic strategy for the management of this rare tumor.

## Introduction

Inflammatory pseudotumor is a rare benign condition of unknown cause. As far as we know, less than 20 cases have been reported in the English literature. It is important to report such rare benign renal tumors in order to determine their reliable characteristics and avoid performing unnecessary nephrectomies that increase the risk of chronic kidney disease. It can be seen in various organs. Originally described in the lungs, a renal location is extremely rare [1]. As inflammatory pseudotumor of the kidney usually mimics renal cell carcinoma, the preoperative diagnosis remains difficult and it is only made through pathological examination of the tumor. We report a case of inflammatory pseudotumor of the kidney; our patient presented with a renal mass and was treated with radical nephrectomy.

## Case presentation

**Figure 1:**
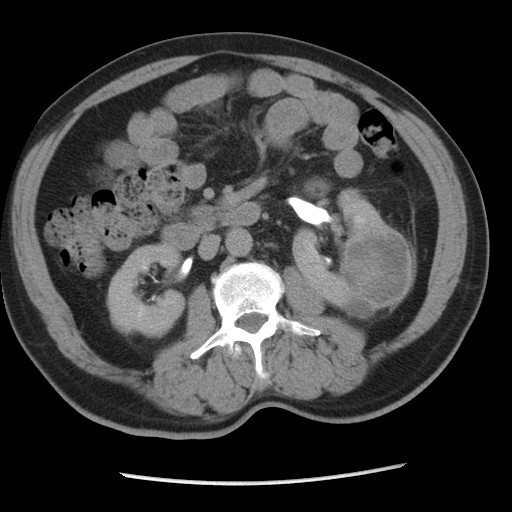
**CT scan showing a huge cystic tumor of the left kidney**.

**Figure 2:**
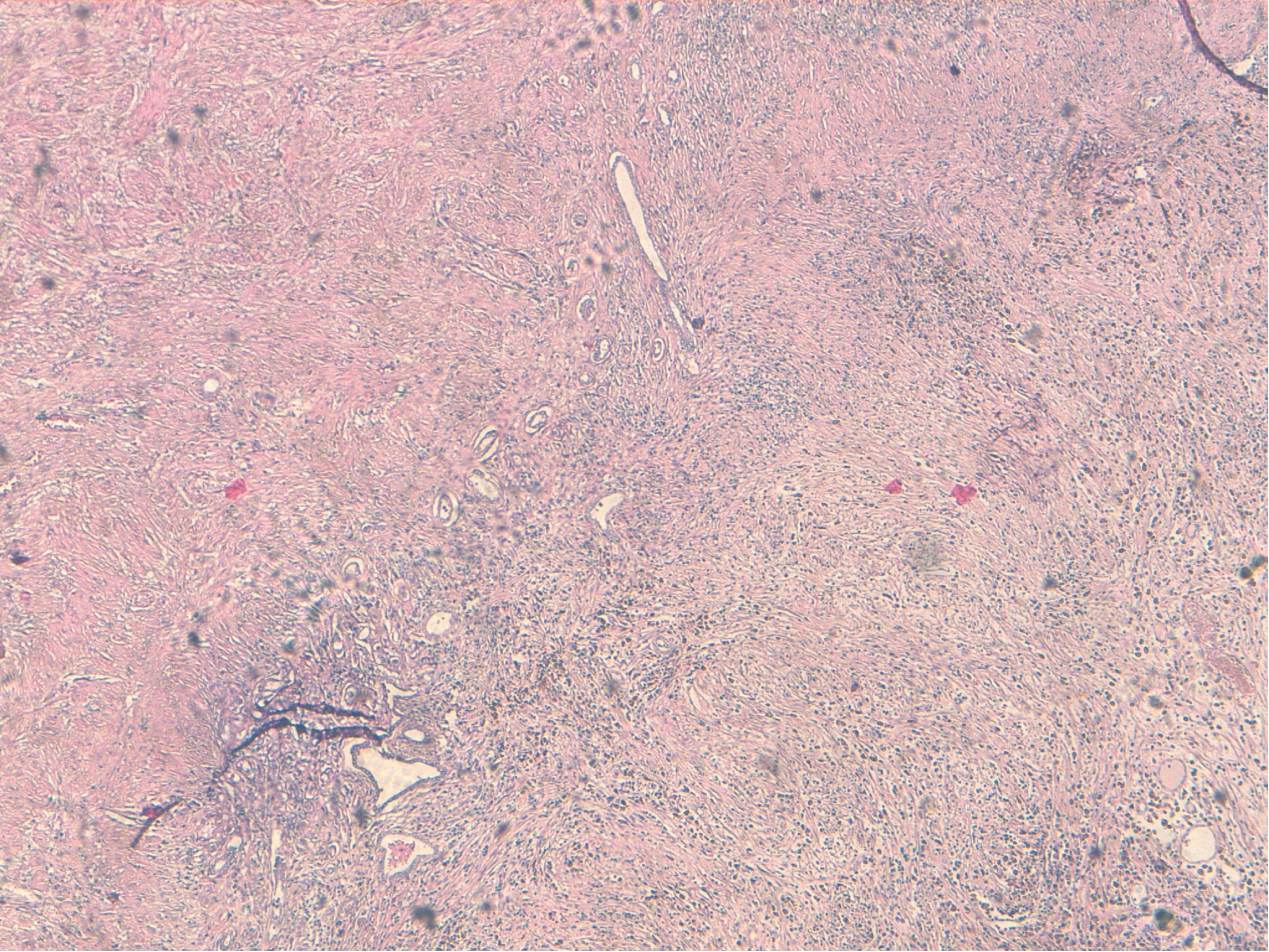
**Photomicrograph showing dense collagen fibrous tissue and inflammation with cellular zone consisting of spindle cells (HES × 5)**.

**Figure 3:**
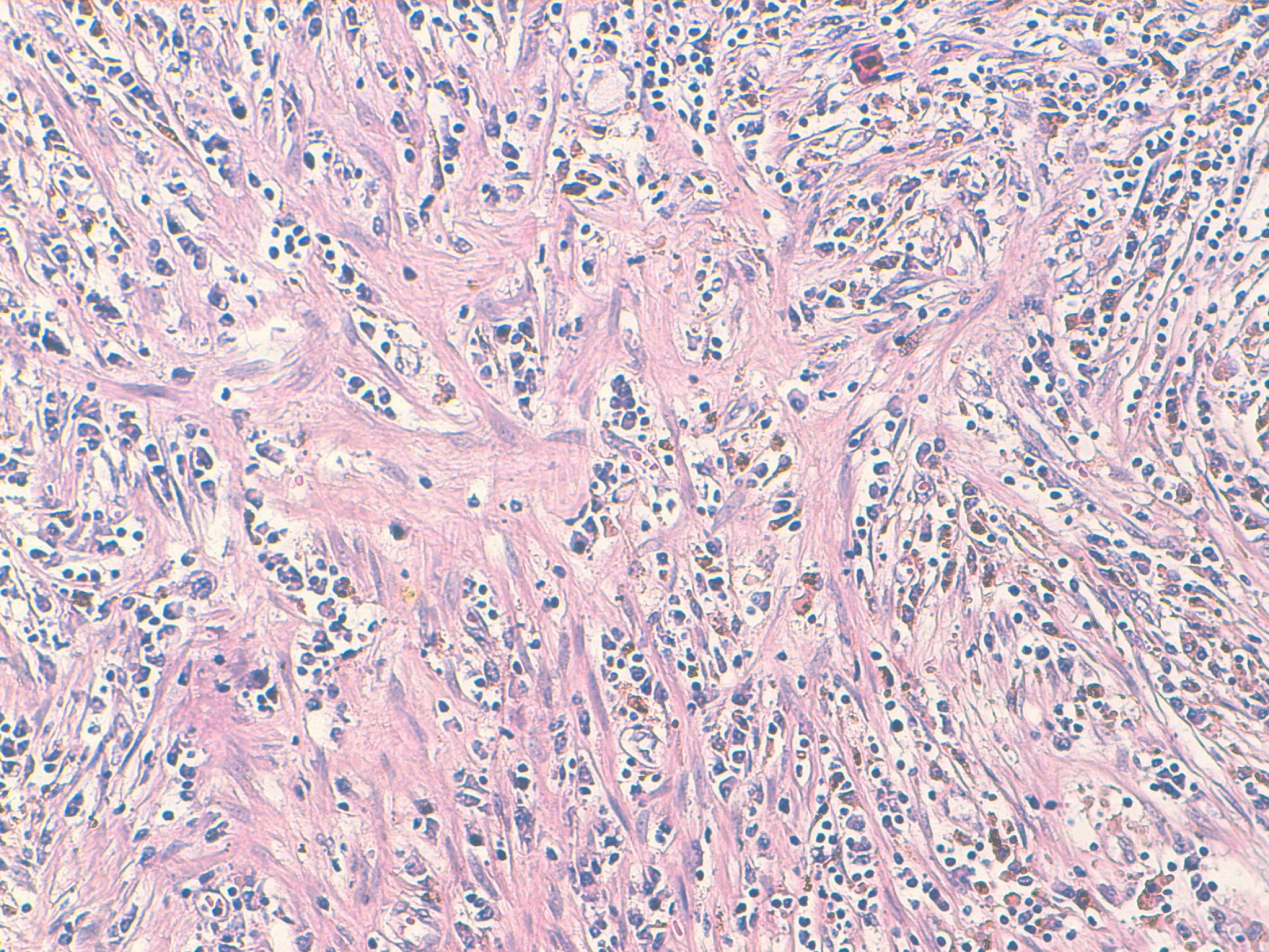
**Photomicrograph showing area of myofibroblastic proliferation with plasma cells and siderophages (HES × 20)**.

A 57-year-old Moroccan man presented with a two-month history of gross hematuria and left lumbar pain. There was no past history of calculous disease or flank pain. He had been smoking 40 cigarettes a day for the past 35 years. The physical and basic paraclinical examinations were normal. Ultrasonography revealed an 8 cm size heterogeneous mass of his left kidney. A contrast-enhanced computed tomography (CT) scan revealed a huge cystic tumor on the left kidney (9.0 × 6.5 × 5.0 cm in size). It was slightly enhanced with contrast, suggesting a malignant tumor such as renal cell carcinoma (Figure 1). Radical nephrectomy was therefore performed under the diagnosis of renal cell carcinoma. Histopathological examination resulted in the lesion being diagnosed as an inflammatory myofibroblastic tumor, in which spindle cells were admixed with variable amounts of extracellular collagen, lymphocytes, plasma cells and siderophages (Figure 2 and 3). Immunostaining was positive for vimentin and HHF-35 and focally positive for smooth muscle actin.

The postoperative course was uneventful and our patient is disease-free after a follow-up of 14 months.

## Discussion

Renal inflammatory pseudotumor (RIP) is very rare. It affects individuals of both sexes and is seen in a wide range of age groups [2]. First described in the lung which is the most common site of involvement, RIP has been described as a benign lesion that mimics malignancy [3]. Differential diagnoses include malignant tumors such as renal cell carcinoma, sarcomatoid renal cell carcinoma, inflammatory fibrosarcoma, malignant fibrous histiocytoma, low grade neurogenic tumor, myxoid leiomyosarcoma and non-malignant tumors such as angiomyolipoma, xanthogranuloma pyelonephritis and plasma cell granuloma [4,5]. The pathogenesis of RIP is still controversial. The inflammatory reaction may be secondary to trauma, surgery, infection or an autoimmune process. Some cases could be related to Epstein-Barr virus infection as some authors reported positivity for Epstein-Barr virus latent membrane protein, especially in the liver and spleen [6,7].

Patients with RIP usually present with lumbar pain and hematuria. Physical examinations and radiological investigations are often inconclusive. RIP can be seen as a hypo- or heterogeneous echoic mass on sonography, a well-defined hypoechoic mass with intratumoral vascularity on enhanced power Doppler sonography, a low-attenuation mass on CT, and hypovascular lesion on magnetic resonance imaging (MRI) [8].

We initially approached our case as renal cell carcinoma due to our patient's symptoms (hematuria and left flank pain) together with CT findings. Some authors reported malignancy associated with inflammatory pseudotumors [9] and it is difficult to make a preoperative diagnosis because symptoms and imaging findings are not specific. It is therefore appropriate to presume the given renal mass to be a renal cell carcinoma and to perform nephrectomy (be it radical or partial). Most diagnoses have been made after surgical intervention [3].

Histological examination is of particular importance to ensure appropriate patient management because RIP can be confused with both reactive process and malignant tumor [10]. RIP consist of a proliferation of spindle cells admixed with various amounts of lymphoplasmacytic infiltrate. Immunohistochemical studies support the myofibroblastic nature of this lesion, with consistent expression of vimentin and smooth muscle actin. These tumors are strongly positive for cluster of differentiation 34 molecule (CD34) reactivity. The architectural appearances vary and have been described as a patternless pattern [10].

## Conclusion

RIP is an extremely rare neoplasm of uncertain biological potential. The preoperative diagnosis remains difficult, despite progress in medical imaging and often requires surgical exploration.

We report a case of RIP treated with radical nephrectomy because the tumor was presumed to be malignant. Histological examination of the specimen confirmed RIP. It is therefore mandatory to carry out good histological examination to make the diagnosis and to assure appropriate patient management.

## Consent

Written informed consent was obtained from the patient for publication of this case report and any accompanying images. A copy of the written consent is available for review by the Editor-in-Chief of this journal.

## Competing interests

The authors declare that they have no competing interests.

## Authors' contributions

AK, MFT and YA have been involved in drafting the manuscript. ET analyzed and interpreted the patient data regarding its oncological features. HE analyzed the pathological features of the specimen. MJE and MHF have given final approval of the version to be published. All authors read and approved the final manuscript.
